# Wine and Olive Oil Phenolic Compounds Interaction in Humans

**DOI:** 10.3390/diseases6030076

**Published:** 2018-09-01

**Authors:** Anna Boronat, Miriam Martínez-Huélamo, Ariadna Cobos, Rafael de la Torre

**Affiliations:** 1 Integrated Pharmacology and Systems Neuroscience Research Group, Neurosciences Research Program, IMIM-Institut Hospital del Mar d’Investigacions Mèdiques, Dr. Aiguader 88, 08003 Barcelona, Spain; aboronat@imim.es (A.B.); mmartinez4@imim.es (M.M.-H.); 2 Department of Experimental and Health Sciences, Universitat Pompeu Fabra (CEXS-UPF), Dr. Aiguader 80, 08003 Barcelona, Spain; acobos2203@gmail.com; 3 CIBER de Fisiopatología de la Obesidad y Nutrición (CIBEROBN, CB06/03/028), Monforte de Lemos 3-5, 28029 Madrid, Spain

**Keywords:** hydroxytyrosol, tyrosol, resveratrol, EVOO, olive oil, RW, red wine, Mediterranean diet

## Abstract

Extra virgin olive oil (EVOO) and red wine (RW) are two basic elements that form part of the so-called Mediterranean diet. Both stand out because of their high phenolic compound content and their potential related health benefits. The present study is focused on the metabolic disposition of resveratrol (RESV), tyrosol (TYR), and hydroxytyrosol (HT) following the consumption of EVOO, RW, and a combination of both. In this study, 12 healthy volunteers consumed a single dose of 25 mL of EVOO, 150 mL of RW, and a combination of both in a crossover randomized clinical trial. Urinary recovery of RESV, TYR, and HT was analysed in urine samples collected over a 6-h period following the intake of each treatment. Higher HT levels were observed following EVOO compared to RW (3788 ± 1751 nmols and 2308 ± 847 nmols respectively). After the combination of EVOO and RW, the recovery of TYR and HT metabolites increased statistically compared to their separate consumption (4925 ± 1751 nmols of TYR and 6286 ± 3198 nmols of HT). EVOO triggered an increase in glucuronide conjugates, while RW intake raised sulfate metabolites. Marginal effects were observed in RESV increased bioavailability after the combination of RW with the fat matrix provided by EVOO.

## 1. Introduction

Research has shown that the Mediterranean diet (MD) reduces the risk of overall mortality and mortality associated with cardiovascular diseases, cancer, Parkinson’s, and Alzheimer’s [1]. Extra virgin olive oil (EVOO) and red wine (RW) represent two of the richest sources of phenolic compounds from the MD. They are thought to be major contributors to the beneficial health effects attributed to the MD. The main phenolic compounds present in EVOO are hydroxytyrosol (HT) and tyrosol (TYR) in the form of their respective secoiridoids oleuropein and ligstroside [2,3,4]. The most well-known polyphenol present in RW is resveratrol (RESV), mainly as its glucoside piceid. Nevertheless, RW contains a wide range of phenolic compounds with biological activities such as gallic acid, syringic acid, hydroxytyrosol, luteolin, and quercitin, among others [5].

RESV (3,4′,5-Trihydroxystilbene) is a natural stilbene present in grape products in two different isomers: the *trans*-isomers (*t*-RESV) and the *cis*-isomers (*c*-RESV). The skin and seeds are the richest parts of the grape. During the RW making process, the skin and the seeds are macerated, facilitating the extraction of RESV. Additionally, alcohol formation during fermentation facilitates this extraction. RESV is well absorbed in the intestine, but its bioavailability is limited because it is rapidly metabolized [6,7]. RESV is a biologically active molecule, which has shown great potential in vitro and pre-clinical studies. The latter is both a chemo preventive and cardio protective agent. In addition, it also offers protection against diabetes, inflammation, and neuro degeneration [8,9]. However, clinical studies are limited and discrepancies have been found in the pre-clinical data. These discrepancies can in part be attributed to disparate doses and poor in vivo bioavailability. Therefore, strategies to increase the bioavailability of RESV are receiving increased attention [10].

HT and TYR have been widely studied in EVOO: both as the main antioxidants and for their potential health benefits. HT is one of the most potent dietary antioxidants. TYR possesses a structure similar to HT, but lacks a hydroxyl group; this results in a lower antioxidant activity compared to HT [11,12]. The EUROLIVE clinical trial provided evidence that olive oil phenolic compounds decreased LDL oxidation, a hallmark in the development of atherosclerosis [13]. As a result, the European Food Safety Agency (EFSA) released a health claim regarding olive oil phenolic compounds. EFSA recommended the ingestion of 5 mg of HT on a daily basis [2]. Furthermore, HT possesses antioxidant and anti-inflammatory properties, which have been shown to inhibit pathological processes involved in cardiovascular and neurodegenerative diseases [14,15]. Moreover, a recent study conducted within the framework of the PREDIMED trial associated high urinary excretion of homovanillyl alcohol (HVALc), a stable metabolite of HT, which provides protection against total mortality and cardiovascular diseases [16].

RW is a source of TYR and to a lesser extent, a source of HT. Both are produced as secondary metabolites of tyrosine during wine fermentation. Despite the low concentrations of HT in RW, significant amounts have been observed after RW ingestion [17]. In this context, it is worthwhile to mention that there is an endogenous formation of HT following ethanol administration. HT is normally produced as a minor metabolite of dopamine oxidative metabolism (also known as DOPET). However, after ethanol administration, dopamine oxidative metabolism is shifted to metabolic pathways, resulting in a significantly higher production of HT in a dose-dependent manner [18]. Nevertheless, the higher recovery of HT after RW consumption could not be explained simply by considering the ethanol-induced formation. Further pre-clinical studies identified TYR as the metabolic precursor of HT [19]. TYR is endogenously bio transformed in humans into HT by means of the isoforms CYP2A6 and CYP2D6 [20]. HT endogenous generation after RW consumption could, in part, explain the beneficial effects derived from moderate wine consumption [17]. Nevertheless, it is worth mentioning that according to the EFSA, a diet containing more than 1.2% of alcohol by volume could not bear any health claims [21].

In the context of the Mediterranean diet, the respective effects of EVOO and RW have been studied extensively. However, to our knowledge, no study investigating the interaction between the metabolic dispositions of their phenolic compounds has yet been conducted. A typical Mediterranean meal includes a serving of EVOO as the fatty component, and a glass of RW. Consumed at the same time, EVOO and RW could interact synergistically, potentiating the bioavailability of their phenolic compounds. The latter would then benefit from the interaction between the hydro-alcoholic properties of RW and the fatty matrix of EVOO, and finally have an impact attenuating the postprandial associated oxidative stress and hyperlipidemia. The aim of the present study was to evaluate RESV, TYR, and HT metabolic disposition after the consumption of EVOO and RW, as well as to assess the potential synergy of its combination on the bioavailability of their phenolic compounds.

## 2. Materials and Methods

### 2.1. Subjects and Study Design

The study consisted of a crossover randomized clinical trial with three different interventions. The interventions consisted of a single administration of 25 mL of EVOO, 150 mL of RW, or the combination of both 25 mL of EVOO and 150 mL of RW. Treatment quantities were equivalent to normal dietary doses of a typical MD. A total of twelve healthy subjects (50% women, 34.0 ± 10.5, BMI = 22.0 ± 3.3 kg/m^2^) participated in the study. The study was explained to participants through verbal and written instructions, and written informed consent was obtained before participation. Volunteers received each treatment in a randomized manner. The study included three experimental sessions in which each intervention was administered under fasting conditions. Each experimental session was preceded by a two-day washout period. The total duration of the study was nine days.

To standardize baseline concentrations of phenolic compounds, subjects were asked to follow a low phenolic content diet, in which participants excluded olive oil and derivates, grapes and derivatives, and all alcoholic beverages from their diet. The low phenolic content diet was followed for two days prior to each intervention and during each experimental session.

On the day of the intervention, subjects consumed one of the three interventions within a period of 5–10 min. Urine was collected in separate fractions, at baseline (−2–0 h), to assess dietary compliance and during 6 h after each dietary intervention (0–6 h). The amount of urine in each fraction was measured, acidified with 6 M HCl, and stored at −20 °C until analysis. The study protocol was approved by the Ethics Committee of Parc de Salut Mar (CEIC-PSMAR) (Spain), and the clinical trial was registered at the International Standard Randomized Controlled Trial Number (NCT03614520).

### 2.2. Red Wine and Extra Virgin Olive Oil

Red wine of the Merlot variety (Cristiari d’Alòs 2014, 13% *v*/*v* ethanol, Costers del Segre, Lleida, Spain) was selected for its high content of resveratrol and piceid (reservatrol-3-β-mono-d-glucoside) in comparison to other red wine varieties [22]. EVOO administrated in the study was produced from arbequina olives obtained directly from olives and extracted solely by mechanical means (Germanor, Les Borges Blanques, Lleida, Spain).

### 2.3. Standards and Reagents

Diethylstilbestrol (internal standard (IS)), β-glucuronidase from *Helix pomatia* type H-2, homovanillyl alcohol (HVALc), 3-(4-hydroxyphenyl)-1-propanol, HT, piceid, *t*-RESV, and Tyr were purchased from Sigma-Aldrich (St Louis, MO, USA). Ethyl glucuronide, HVALc-glucuronide, HT-acetate-sulfate, HT-glucuronide, HT-3-sulfate, TYR-glucuronide, and TYR-sulfate, as well as the internal standards ethyl-glucuronide-d_5_, 4-(3-hydroxypropyl) phenyl glucuronide, HT-D_3_, and HT-1-sulfate, were purchased from Toronto Research Chemicals Inc. (Toronto, ON, Canada). Ammonium iodide (NH_4_I), formic acid (H-COOH), hydrochloric acid (HCl), 2-mercaptoethanol, phosphoric acid, sodium acetate, sodium chloride (NaCl), sodium hydroxide (NaOH), and sodium metabisulfite were purchased from Merck (Darmstadt, Germany). Acetonitrile, ethyl acetate, and methanol were supplied by Scharlab SL (Barcelona, Spain), while *N*-methyl-*N*-trimethylsilyltrifluoroacetamide (MSTFA) was supplied by Macherey–Nagel (Düren, Germany). Dihydroresveratrol and *c*-RESV were prepared from *t*-RESV as previously reported [3]. Oasis HLB 3cc Vac Cartridges (60 mg) (WAT094226) for solid-phase extraction were purchased from Waters Corporation (Milford, MA, USA). Ultrapure water (Milli-Q) was obtained from a Millipore system (Millipore, Bedford, MA, USA) and blank human urine from volunteers after three days of a diet restricted in alcohol, grape, and olive derivates.

### 2.4. Extraction and Analysis of Resveratrol in Red Wine and Urine Samples

#### 2.4.1. Red Wine

RESV and piceid content in RW was measured by gas chromatography coupled to mass spectrometry (GC-MS) after a liquid-liquid extraction, as previously described [6]. In short, 1 mL of diluted red wine (1:10 in water) was extracted with 5 mL ethyl acetate in amber glass tubes (to avoid *t*-RESV conversion to its *cis* form). After being shaken for 30 min, samples were centrifuged for 5 min at 300 g and the supernatant was transferred to another amber tube to be evaporated until dry by a sample concentrator (Caliper Life Sciences, Waltham, Massachusetts, MA, USA) at 30 °C under a stream of nitrogen. After 1 h in an oven at 50 °C, the residue was derivatized with 75 µL of MSTFA: NH4I:2-mercaptoethanol reaction mixture (2 g NH_4_I and 5 mL of 2-mercaptoethanol per liter of MSTFA) for 30 min at 60 °C. Calibration curves were prepared by adding different concentrations of *c*- and *t*-RESV and piceid (100–1000 µg/L) to water (10 mL) and extracted in the same way as red wine samples. Finally, 2 µL was injected into the gas chromatograph.

#### 2.4.2. Urine Samples

Urine was subjected to a hydrolysis procedure previously described by our working group [3]. Aliquots of 1 mL of diluted urine (1:10 in water) were spiked with 10 µL of IS (containing 10 µg/mL of diethylstilbestrol), 100 µL of sodium metabisulfite, 1 mL acetate buffer 0.1 M pH 5.2, and 25 µL of β-glucuronidase. The samples were incubated at 37 °C overnight. After incubation, 1 mL of NaOH was added to neutralize the hydrolysis process. For the extraction of the phenolic compounds, 0.5 mL of a saturated solution of NaCl and 4 mL of acetonitrile-ethyl acetate mixture (1:4 *v*/*v*) were added. Samples were mixed for 30 min, centrifuged for 5 min at 300 g, and the organic phase was extracted and evaporated until dry. After 1 h in an oven at 50 °C, the residue was derivatized with 75 µL of MSTFA: NH4I:2-mercaptoethanol reaction mixture for 30 min at 60 °C and 2 µL was injected into the gas chromatograph.

For the preparation of the calibration curves, 1 mL of diluted urine (1:10 in water) was spiked with an increasing concentration of *c*- and *t*-RESV and dihydro-RESV (10–200 ng/mL), and subjected to the extraction procedure exactly in the same way as the samples.

The extracted samples were analyzed using an Agilent Technologies (Santa Clara, CA, USA) 6890 N gas chromatograph coupled to a 5973 mass-selective detector. For chromatographic separation, a 5% phenyl-dimethyl-polysiloxane Zebron™ (Torrance, CA, USA) fused-silica capillary column (15 m × 0.25 mm i.d., 0.25 µm film thickness) was used. The split injection mode using helium as a carrier gas (0.9 mL/min) was applied. The temperatures of the injector and transfer line were set at 280 °C. Gas chromatographic conditions were as follows: initial oven temperature at 80 °C, raised by 20 °C/min to 200 °C, then by 10 °C/min to 300 °C, and maintained at 300 °C for 3 min. The mass spectrometer was operated in the selected ion monitoring mode (SIM) and had an electron impact of 70 eV. Ions at *m*/*z* 444 (RESV and piceid), *m*/*z* 179 (dihydro-RESV), and *m*/*z* 412 (diethylstilbestrol), were selected for the quantitative analysis. For the confirmation of the compounds, the ions chosen were *m*/*z* 445 for RESV and piceid, *m*/*z* for 446 dihydro-RESV, and *m*/*z* 397 and 383 for diethylstilbestrol.

### 2.5. Extraction and Analysis of Hydroxytyrosol in Extra Virgin Olive Oil, Red Wine and Urine Samples

#### 2.5.1. Extra Virgin Olive Oil

TYR and HT content in EVOO were quantified by liquid chromatography coupled with tandem mass spectrometry (LC-MS/MS) after a triple liquid-liquid extraction, as previously described [23]. Briefly, aliquots of 1 mL of olive oil were spiked with 10 µL of IS (containing 100 µg/mL of HT-D_3_ and 3-(4-hydroxyphenyl)-1-propanol). A first liquid-liquid extraction was performed with 10 mL of methanol/water solution (80:20, *v*/*v*) containing 1 mM of ascorbic acid to avoid phenol degradation during the process. Tubes were shaken for 60 min, and then centrifuged (2000 g, 5 min). The organic phase was transferred into a new tube and evaporated under a nitrogen stream at 30 °C to a final remaining volume of 2 mL of an aqueous extract of olive oil. Thereafter, metabisulfite was added to the samples to prevent oxidation. To hydrolyze all the conjugated forms of TYR and HT, samples were incubated at 37 °C for 30 min with HCl (1.5 mmol/tube), to mimic gastrointestinal conditions during digestion. Following that, a liquid-liquid extraction was performed by adding 4 mL of a mixture of ethyl acetate and acetonitrile (4:1 *v*/*v*) shaking for 30 min and centrifuging (2000 g, 5 min). The organic phase was transferred into a new tube and the liquid-liquid extraction was repeated, finally combining both organic phases into the same tube and evaporating the mixture until completely dry. Extracts were reconstituted with 100 µL of mobile phase containing (80% A: 20% B) and injected into the LC-MS/MS. The composition of mobile phase A was 0.01% of ammonium acetate (pH 5) in water; mobile phase B was pure methanol. Calibration curves were prepared by adding standards of TYR and HT to 1 mL of refined oil. All the samples were analyzed in triplicate.

Samples were analyzed using an Agilent Technologies 6410 Triple Quad (Santa Clara, CA, USA). The separation was carried out with an Acquity UPLC^®^ BEH C18 column (Waters, Milford, MA, USA) with a 1.7 μm particle size, 3 mm × 100 mm (Waters, Milford, MA, USA). The injection volume was 10 μL and the ion source operated in negative ionization mode.

#### 2.5.2. Red Wine

Red wine content of TYR and HT was determined by LC-MS/MS after a simple dilution. Briefly, red wine samples were diluted 40 times with mobile phase (65% A: 35% B) spiked with 10 µL of IS (containing 10 µg/mL of hydroxytyrosol-D_3_ and 3-(4-hydroxyphenyl)-1-propanol). Calibration curves were prepared by adding standards of TYR and HT to pure water. All samples were analyzed in triplicate. The composition of mobile phase A was 0.01% of ammonium acetate (pH 5) in water; mobile phase B was pure methanol. Samples were analyzed using an Agilent Technologies 6410 Triple Quad (Santa Clara, California, CA, USA). The separation was carried out with an Acquity UPLC^®^ BEH C18 column 1.7 μm particle size, 3 mm × 100 mm (Waters, Milford, MA, USA). The injection volume was 10 μL and the ion source operated in negative ionization mode.

#### 2.5.3. Urine Samples

The quantification of urinary levels of HT and TYR free forms and their metabolites was performed using a solid-phase extraction and following the method previously described [24,25]. The method was capable of detecting the free forms HT, TYR, and HVALc; the sulfate conjugates HT-sulfate, TYR-sulfate, and HT-acetate-sulfate; and the glucuronide conjugates HT-glucuronide, TYR-glucuronide, and HVALc-glucuronide. Shortly thereafter, aliquots of 0.5 mL of the samples were diluted with 0.5 mL of purified water and spiked with 10 µL of internal standard (containing 10 µg/mL of HT-D_3_, 3-(4-hydroxyphenyl)-1-propanol, 4-(3-hydroxypropylphenyl) glucuronide and HT-1-sulfate) and stabilized with 1 mL of phosphoric acid 4%. Thereafter, samples were submitted to a solid-phase extraction by means of Oasis HLB columns. Samples were loaded into the cartridges, and the cartridges were then washed with 2 mL of purified water. Finally, the compounds of interest were eluted by adding 2 mL of methanol to the cartridges. Subsequently, the methanol was evaporated until dry using nitrogen (29 °C, 10–15 psi). Finally, the dried extracts were reconstituted in 100 µL of a mixture of mobile phases (91% A/9% B *v*/*v*), transferred into HPLC vials, and analyzed by LC-MS/MS.

Identification and quantification of HT and TYR metabolites was performed using an Agilent 1200 series HPLC system (Agilent technologies, Santa Clara, California, CA, USA) coupled to a triple quadrupole (6410 Triple Quad LC/MS; Agilent) mass spectrometer with an electrospray interface. The chromatographic separation was carried out with an Acquity UPLC^®^ BEH C18 column with a 1.7 μm particle size, 3 mm × 100 mm (Waters, Milford, MA, USA) maintained at 40 °C. The composition of mobile phase A was 0.01% of ammonium acetate (pH 5) in water; mobile phase B was pure methanol. The injection volume was 10 μL and the ion source operated in negative ionization mode.

### 2.6. Ethyl Glucuronide Quantification

Ethyl glucuronide concentration in urine was used as a marker of alcohol abstinence and compliance with the dietary recommendations before each intervention. To determine urinary concentrations of ethyl glucuronide, aliquots of 30 μL were mixed with 10 μL of IS mix solution (containing 10 μg/mL ethyl-glucuronide-d_5_ and ethyl-sulfate-d_5_) and 110 μL of 0.1% formic acid solution in water. The identification and the quantification of ethyl glucuronide were carried out using an Agilent 1200 series HPLC system (Agilent technologies) (Santa Clara, California, CA, USA) coupled to a triple quadrupole (6410 Triple Quad LC/MS; Agilent) mass spectrometer with an electrospray interface. To perform the chromatographic separation, an Acquity UPLC^®^ BEH C18 column with a 1.7 μm particle size, 3 mm × 100 mm (Waters, Milford, MA, USA) was used. The composition of mobile phase A was 0.1% (*v*/*v*) formic acid in water, and mobile phase B was 0.1% (*v*/*v*) formic acid in acetonitrile.

### 2.7. Statistical Analysis

Primary outcomes were HT, TYR, and RESV urinary recovery. Sample size calculation was based on HT urinary recovery and indicated that a total of 12 volunteers was enough to detect a difference of 1000 nmol of HT with a power of 90% and α = 0.05. All the results were subjected to a normality test prior to the statistical analysis and then to one-way analysis of variance (ANOVA) with the Bonferroni post hoc test in the case of homogeneity of variances and T3 Dunnett when the variances were not homogenous. The results were reported as the mean ± standard deviation (SD). Differences at *p* < 0.05 were considered statistically significant. SPSS software (Version 18.0, Japan Inc., Tokyo, Japan) was used for data analysis.

## 3. Results

### 3.1. Phenolic Content in Extra Virgin Olive Oil and Red Wine

In order to characterize the principal phenolic compounds of the interventions, EVOO and RW were analyzed in triplicate. Table 1 shows the concentration observed for RESV and its isomers, corresponding to RW, and the concentration of HT and TYR of RW and EVOO treatments.

RW contained a concentration of 2.4 ± 0.1 mg/L of *t*-RESV, while the concentration of *c*-RESV was found to be 3.0 ± 0.4 mg/L. Regarding piceid, *t*-piceid was determined at a concentration of 4.9 ± 0.2 mg/L and its isomer *cis* obtained a concentration of 3.0 ± 0.5 mg/L. HT and TYR were also analyzed in RW with concentrations of 1.5 ± 0.1 and 35.0 ± 1.0 mg/L, respectively, and in EVOO, HT was detected at a concentration of 19.8 ± 1.9 mg/L and TYR at 24.1 ± 2.8 mg/L.

Therefore, for RW treatments (150 mL), about 0.36 mg of *t*-RESV, 0.45 mg of *c-*RESV, 0.74 mg of *t*-piceid, 0.45 mg of *c*-piceid, 0.22 mg of HT, and 5.25 mg of TYR were administered. Regarding EVOO, 25 mL of the dose administrated corresponds to 0.50 mg of HT and 0.60 mg of TYR.

### 3.2. Quantification of Phenolic Compounds in Urine

#### 3.2.1. Baseline

Diet compliance was assessed by the baseline analysis of urine samples collected 2 h before the beginning of the intervention (−2 to 0 h). Volunteers followed the washout recommendations perfectly since no RESV was observed in baseline urine samples. In terms of HT, TYR, and metabolites, as they are endogenous compounds, traces could be observed in baseline samples, but these were not attributed to the diet contribution.

#### 3.2.2. Resveratrol

**Figure 1:**
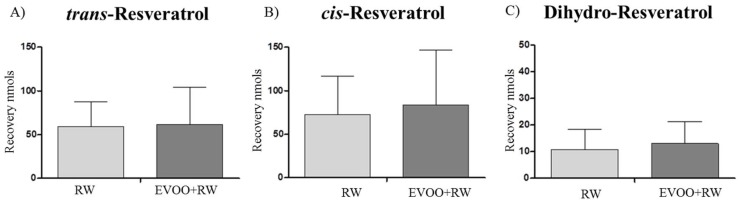
RESV urinary recovery (nmol) from 0 to 6 h after RW and RW + EVOO of (**A**) *t-*RESV; (**B**) *c-*RESV; and (**C**) Dihydro-RESV. Data expressed as mean ± SD.

Urinary amounts of RESV and its isomers were analyzed in the three interventions, both at baseline and 6 h after consumption. Table 2 shows the concentration of the phenolic compounds found during the study. Only when RW was administered, RESV and its isomers were identified (Figure 1). Urinary recovery of *t*-RESV after 6 h in the RW treatment was 59.2 ± 28.7 nmol, while after RW + EVOO, the concentration of this compound reached 61.7 ± 42.4 nmol. Similar results were obtained with dihydro-RESV, which increased its concentration following RW + EVOO treatment compared with RW (13.0 ± 8.3 nmol RW + EVOO vs. 10.9 ± 7.5 nmol RW). Regarding *c*-RESV, the compound also presented a greater increase in RW + EVOO treatment, although none of the interventions presented significant differences, probably due to high interindividual differences.

#### 3.2.3. Hydroxytyrosol

**Figure 2:**
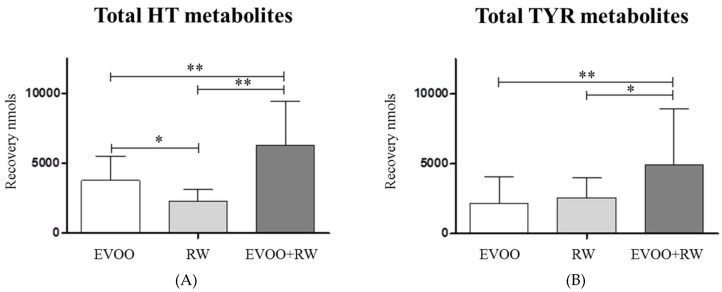
HT and TYR urinary recovery (nmol) from 0 to 6 h after EVOO, RW, and RW+EVOO (*n* = 12) of (**A**) Total HT (HT-glucuronide + HT-sulfate + HT-acetate-sulfate + free HT + HVALc free + HVALc glucuronide) and (**B**) Total TYR (Tyrosol-glucuronide + TYR-sulfate + free Tyrosol). Data expressed as mean ± SD. * *p* < 0.05; ** *p* < 0.01.

HT and its metabolites were analyzed by LC-MS/MS, obtaining a total of nine metabolites found in urine after the three interventions (Table 3). The analytical method included the quantification of the free forms and phase II metabolites conjugated with sulfate and glucuronide. Figure 2 shows the sum of HT metabolites (Figure 2A) and TYR metabolites (Figure 2B) after each intervention. Figure 2A shows differences in HT recovery between the three interventions. EVOO + RW had the highest recovery. Similar results were obtained regarding TYR metabolites, and significant differences were obtained between EVOO + RW and RW and between EVOO + RW and EVOO, but no difference was observed in TYR recovery between EVOO and RW.

**Figure 3:**
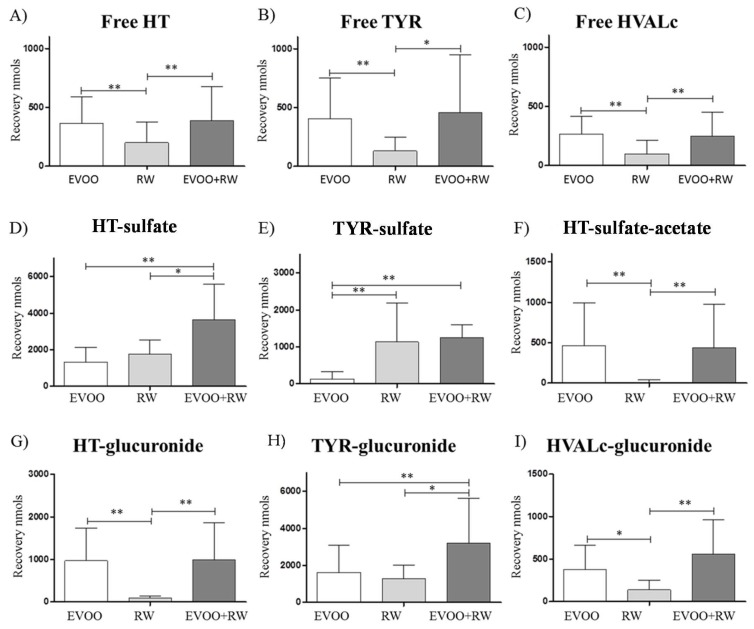
HT and TYR free forms, sulfate, and glucuronide metabolites urinary recovery (nmol) from 0 to 6 h after EVOO, RW, and RW + EVOO (*n* = 12) of (**A**) Free HT; (**B**) Free TYR; (**C**) Free HVALc; (**D**) HT-sulfate; (**E**) TYR-sulfate; (**F**) HT-sulfate-acetate; (**G**) HT-glucuronide; (**H**) TYR-glucuronide; and (**I**) HVALc-glucuronide. Data expressed as mean ± SD. * *p* < 0.05; ** *p* < 0.01.

When analyzing the different metabolic pathways in more depth after each intervention, it was observable that free forms were present at low concentrations (between 10–15% of the total), while the sulfate and glucuronide conjugates were the most abundant. HT-sulfate increased following both interventions, whereas TYR-sulfate was increased exclusively after RW; on the contrary, HT-sulfate-acetate was only present after EVOO. When comparing glucuronides, HT-glucuronide was only generated after the EVOO treatment, and HVALc-glucuronide was detectable after RW, but its major contributor was EVOO. Finally, TYR-glucuronide increased equally after both interventions (Figure 3).

### 3.3. Ethyl Glucuronide

Ethyl glucuronide at baseline was undetectable, confirming that volunteers followed an alcohol-free diet. Ethyl glucuronide recovery was almost identical after RW intervention (19.0 ± 9.5 µmols) and RW + EVOO intervention (19.3 ± 7.6 µmols), while it was undetectable after EVOO.

## 4. Discussion

It is the first time in humans that the interaction between RW and EVOO is evaluated in terms of the metabolic disposition of the main phenolic compounds of both Mediterranean food components. Here, we report that recoveries of phenolic compounds from RW and EVOO are altered when combining both foods. There is a recovery of HT and related compounds that doubles the expected concentrations taking into consideration amounts of HT present in RW and EVOO at the doses administered. On the other hand, a non-significant increase of resveratrol-related compounds is observed when combining RW and EVOO.

The study of the interaction of both foods is of relevance since phenolic compounds typically have a poor bioavailability, being very much dependent on the matrix in which they are present [6,10,26]. The mixture of a fatty matrix and a hydro-alcoholic one may influence its bioavailability. Some preliminary results suggest that this would be the case and that the interaction may result in beneficial health effects [27].

In the case of HT, we previously reported that there is an interaction between alcohol and phenolic components of RW, in particular, TYR. Alcohol, on the one hand, interacts with dopamine oxidative metabolism, promoting a shift in its metabolic pathways. A minor pathway from DOPAL (3,4-Dihydroxyphenylacetaldehyde) to DOPET (3,4-dihydroxyphenylethanol, also known as HT) becomes more apparent in the presence of alcohol. In humans, we have demonstrated that DOPET (HT) generation is alcohol dose dependent [18]. Similarly there is an increased synthesis of TYR via tyrosine metabolic disposition in an analogous way, as described for the dopamine and ethanol interaction. Although there is a contribution of dopamine and tyrosine when alcohol is consumed in the formation of HT and TYR, this does not suffice to explain recoveries of HT and TYR [19]. We demonstrated in vivo, in animal models, and in vitro, in human liver biopsies, that the most likely explanation for higher recoveries of both phenolic compounds is the biotransformation of TYR to HT, a reaction regulated by the polymorphic enzymes CYP2D6 and CYP2A6 [20]. Ethanol contributes to this reaction by increasing TYR bioavailability and then favoring the biotransformation reaction. Here, we demonstrate for the first time that the contribution of this reaction leading to HT formation by RW is quite substantial when compared to recoveries after EVOO. The dose of total HT contained in RW represents 44% of the one contained in EVOO. Nevertheless, the total HT recovery following RW represents 60% of the recovery after EVOO. This observation confirms an endogenous formation of HT following RW consumption.

The metabolic disposition of HT has been reviewed recently [2]. When comparing the metabolic pathways of HT for EVOO and RW, a higher recovery of unaltered HT is observed after EVOO. Therefore the impact of HT from EVOO when compared to RW in terms of biological effects would be superior. Nonetheless, when looking at metabolic pathways, it is apparent that HT-sulfate and TYR-sulfate recoveries are higher after RW than after EVOO, and 60% of the total metabolites were sulfate conjugates in the case of RW compared with the 32% in the case of EVOO. This is of relevance since these metabolites have been reported to be biologically active, specifically preventing the effects of oxidized cholesterol, and not the corresponding glucuronide metabolite [28].

Regarding resveratrol, marginally higher concentrations of *t*-RESV, *c-*RESV, and dihydro-RESV are observed after RW combined with EVOO, suggesting that resveratrol bioavailability is slightly increased in the presence of EVOO.

## Figures and Tables

**Table 1:** Phenolic content of EVOO, RW, and administered doses.

Treatment	Concentration (mg/L)						Dose Administered (mL)	Dose Administered (mg)					
	*t*-RESV	*c*-RESV	*t*-Piceid	*c*-Piceid	HT	TYR		*t*-RESV	*c*-RESV	*t*-Piceid	*c*-Piceid	HT	TYR
**EVOO**	0	0	0	0	19.8 ± 1.9	24.1 ± 2.8	25	0	0	0	0	0.50	0.60
**RW**	2.4 ± 0.1	3.0 ± 0.4	4.9 ± 0.2	3.0 ± 0.4	1.5 ± 0.1	35.0 ± 1.0	150	0.36	0.45	0.74	0.45	0.22	5.25
**EVOO + RW**	NA	NA	NA	NA	NA	NA	25 + 150	0.36	0.45	0.74	0.45	0.72	5.85

Phenolic composition of EVOO and RW and the equivalent doses administered in the study. Data expressed as mean ± SD.

**Table 2:** RESV urinary recovery (nmol) from 0 to 6 h after treatments.

Phenolic Compound (nmols)	EVOO	RW	EVOO + RW
*t-*RESV	0.0 ± 0.0	59.2 ± 28.7 ^aa^	61.7 ± 42.4 ^aa^
*c-*RESV	0.0 ± 0.0	72.8 ± 44.3 ^aa^	83.8 ± 62.6 ^aa^
Dihydro-RESV	0.0 ± 0.0	10.9 ± 7.5 ^aa^	13.0 ± 8.3 ^aa^

Urinary excretion 0–6 h of *t*-RESV, *c-*RESV, and dihydro-RESV after EVOO, RW, and EVOO + RW (*n* = 12). Data expressed as mean ± SD. ^aa^ *p* < 0.01 versus EVOO.

**Table 3:** HT, TYR, and metabolites urinary recovery (nmol) from 0 to 6 h after treatments.

Phenolic Compound (nmols)	EVOO	RW	EVOO + RW
Total HT	3788 ±1751	2308 ± 847 ^a^	6286 ± 3198 ^aa bb^
Total TYR	2180 ± 1917	2567 ± 1468	4925 ± 3993 ^aa b^
Free HT	367 ± 221	201 ± 173 ^aa^	386 ± 289 ^bb^
Free TYR	404 ± 346	132 ± 114 ^aa^	460 ± 490 ^b^
Free HVALc	269 ± 145	110 ± 118 ^aa^	247 ± 205 ^bb^
HT-sulfate	1336 ± 795	1767 ± 787	3655 ± 1926 ^aa b^
TYR-sulfate	138 ±194	1133 ± 1052 ^aa^	1252 ± 1190 ^bb^
HT-acetate-sulfate	465 ± 528	11.2 ± 30.9 ^aa^	436 ± 543 ^bb^
HT-glucuronide	974 ± 766	90.5 ± 56.3 ^aa^	1000 ± 856 ^bb^
TYR-glucuronide	1639 ± 1438	1301 ± 720	3215 ± 2421 ^aa b^
HVALc-glucuronide	376 ± 284	139 ± 114 ^a^	563 ± 401 ^bb^

Urinary excretion 0–6 h of total HT metabolites, total TYR metabolites, and single metabolites after EVOO, RW, and EVOO + RW (*n* = 12). Data expressed as mean ± SD. ^a^ *p* < 0.05, ^aa^ *p* < 0.01 versus EVOO; ^b^ *p* < 0.05, ^bb^ *p* < 0.01 versus RW. Total HT = HT-glucuronide + HT-sulfate + HT-acetate-sulfate + free HT + HVALc free + HVALc glucuronide); Total Tyrosol = Tyrosol-glucuronide + TYR-sulfate + free Tyrosol.

## Abbreviations

NH_4_I	ammonium iodide;
EFSA	European Food Safety Agency;
EVOO	extra virgin olive oil;
GC-MS	gas chromatography coupled to mass spectrometry;
H-COOH	formic acid;
HVALc	homovanillyl alcohol;
HCl	hydrochloric acid;
HT	hydroxytyrosol;
IS	internal standard;
LC-MS/MS	liquid chromatography coupled to tandem mass spectrometry;
MD	Mediterranean diet;
MSTFA	*N*-methyl-*N*-trimethylsilyltrifluoroacetamide;
RW	red wine;
RESV	resveratrol;
NaCl	sodium chloride;
NaOH	sodium hydroxide;
TYR	tyrosol.
